# Distinct Gene Expression Patterns of Calcium Channels and Related Signaling Pathways Discovered in Lymphomas

**DOI:** 10.3389/fphar.2022.795176

**Published:** 2022-05-24

**Authors:** Shawna R. Stanwood, Lauren C. Chong, Christian Steidl, Wilfred A. Jefferies

**Affiliations:** ^1^ Michael Smith Laboratories, University of British Columbia, Vancouver, BC, Canada; ^2^ Vancouver Prostate Centre, Vancouver General Hospital, Vancouver, BC, Canada; ^3^ Centre for Blood Research, University of British Columbia, Vancouver, BC, Canada; ^4^ Department of Microbiology and Immunology, University of British Columbia, Vancouver, BC, Canada; ^5^ Centre for Lymphoid Cancer, British Columbia Cancer Research Institute, Vancouver, BC, Canada; ^6^ Lymphoid Cancer Research, British Columbia Cancer Research Institute, Vancouver, BC, Canada; ^7^ Department of Pathology and Laboratory Medicine, University of British Columbia, Vancouver, BC, Canada; ^8^ Djavad Mowafaghian Centre for Brain Health, University of British Columbia, Vancouver, BC, Canada; ^9^ Department of Medical Genetics, University of British Columbia, Vancouver, BC, Canada; ^10^ Department of Urological Sciences, University of British Columbia, Vancouver, BC, Canada; ^11^ Department of Zoology, University of British Columbia, Vancouver, BC, Canada

**Keywords:** calcium channel, lymphoma, leukaemia, signaling pathway, sequencing

## Abstract

Cell surface calcium (Ca^2+^) channels permit Ca^2+^ ion influx, with Ca^2+^ taking part in cellular functions such as proliferation, survival, and activation. The expression of voltage-dependent Ca^2+^ (Ca_V_) channels may modulate the growth of hematologic cancers. Profile analysis of Ca^2+^ channels, with a focus on the Ca^2+^ release-activated Ca^2+^ (CRAC) and L-type Ca_V_ channels, was performed on RNA sequencing data from lymphoma cell lines and samples derived from patients with diffuse large B cell lymphoma (DLBCL). Ca_V_1.2 expression was found to be elevated in classical Hodgkin lymphoma (CHL) cell lines when compared to other B cell lymphoma cell lines. In contrast, CHL exhibited reduced expression of ORAI2 and STIM2. In our differential expression analysis comparing activated B cell-like DLBCL (ABC-DLBCL) and germinal centre B cell-like DLBCL (GCB-DLBCL) patient samples, ABC-DLBCL revealed stronger expression of Ca_V_1.3, whereas Ca_V_1.1, Ca_V_1.2, and Ca_V_1.4 showed greater expression levels in GCB-DLBCL. Interestingly, no differences in ORAI/STIM expression were noted in the patient samples. As Ca^2+^ is known to bind to calmodulin, leading to calcineurin activation and the passage of nuclear factor of activated T cells (NFAT) to the cell nucleus, pathways for calcineurin, calmodulin, NFAT, and Ca^2+^ signaling were also analyzed by gene set enrichment analysis. The NFAT and Ca^2+^ signaling pathways were found to be upregulated in the CHL cell lines relative to other B cell lymphoma cell lines. Furthermore, the calmodulin and Ca^2+^ signaling pathways were shown to be downregulated in the ABC-DLBCL patient samples. The findings of this study suggest that L-type Ca_V_ channels and Ca^2+^-related pathways could serve as differentiating components for biologic therapies in targeted lymphoma treatments.

## Introduction

Hodgkin lymphoma, non-Hodgkin lymphoma (NHL), and leukemia collectively constitute 5.7% of all new cases of cancer (Sung et al., 2021). The ability to conduct expression profiling of genes has greatly contributed to knowledge pertaining to leukemia and lymphoma in terms of categorization of subtypes (Hoefnagel et al., 2005; Bobée et al., 2020), disease aggressiveness (Glas et al., 2005), and relapse (Yeoh et al., 2002). Differences in the expression of subunits of ion channels, including potassium (K^+^) channels, sodium (Na^+^) channels, and calcium (Ca^2+^) channels, have been noted in relapsed follicular lymphoma compared to counterparts who have not relapsed (Magi et al., 2019). The Ca^2+^ release-activated Ca^2+^ (CRAC) channel, a well-characterized example of a Ca^2+^ channel, consists of stromal interacting molecule (STIM) and ORAI proteins (Putney, 2018). Orai3 expression has been shown to be elevated in leukemia/multiple myeloma cell lines sensitive to tipifarnib when compared to a resistant myeloma cell line (Yanamandra et al., 2011). Furthermore, upregulation and downregulation of voltage-dependent Ca^2+^ (Ca_V_) channel expression have been established for leukemia and lymphoma, along with many additional kinds of cancer (by publicly accessible data from microarrays), suggesting that the channels are engaged in the development of cancer (Wang et al., 2015; Phan et al., 2017).

Ca^2+^ channels are known to be positioned at the plasma membrane of the cell (Omilusik et al., 2013). Once Ca^2+^ is in the cell, it can guide proliferation, homeostasis, differentiation, survival, and activation by taking part in intracellular pathways (Omilusik et al., 2013). In T lymphocytes, for example, Ca^2+^ can bind to calmodulin, enabling the latter to activate the enzyme calcineurin once bound (Omilusik et al., 2013). Nuclear factor of activated T cells (NFAT) is dephosphorylated by calcineurin, leading to NFAT moving into the cell nucleus (Omilusik et al., 2013). NFAT has long been known to bind to the interleukin-2 (IL-2) promoter with activating protein-1 (AP-1) (Rao et al., 1997). Several other “transcriptional partners” of NFAT include T-bet at the 5’ enhancer for interferon (IFN)-γ and IFN-regulatory factor 4 (IRF4) at the promoter for interleukin-4 (IL-4) (Macian, 2005).

Considering the involvement of Ca^2+^ in lymphocyte processes, it would be useful to study Ca^2+^ channel expression more thoroughly in lymphoma. In order to accomplish this objective, patient samples and cell lines were assessed in the current study. This investigation focuses on diagnostic tissue from patients with diffuse large B cell lymphoma (DLBCL) and cell lines representing diverse types of lymphoma. Expanding on the calmodulin/calcineurin/NFAT signaling pathway, several pathways from BioCarta and the Kyoto Encyclopedia of Genes and Genomes (KEGG) were also examined. Here, we present expression profiles relating to Ca^2+^ channels - the CRAC and Ca_V_ channels in particular - and these pathways. This characterization of expression may aid in cultivating a comprehensive understanding of the molecular basis of lymphoma.

## Materials and Methods

### RNA Sequencing Data

RNA sequencing data were generated at the British Columbia Cancer Research Centre (BC Cancer) from 44 lymphoma cell lines representing multiple pathologies. These datasets were generated at multiple time points and were consolidated for analysis in this study (Table 1).

**TABLE 1:** Sequencing conditions used for RNA sequencing of lymphoma cell lines. Cell lines of various pathologies were included for analysis.

Cell Line	Read Length	Sequencer	Pathology	Source
HDLM-2	75bp	HiSeq	Classical Hodgkin Lymphoma	BC Cancer
HDMYZ	151bp	MiSeq	Classical Hodgkin Lymphoma	BC Cancer
KM-H2	50bp	GA	Classical Hodgkin Lymphoma	BC Cancer
L-1236	75bp	HiSeq	Classical Hodgkin Lymphoma	BC Cancer
L-428	50bp	GA	Classical Hodgkin Lymphoma	BC Cancer
L-540	75bp	HiSeq	Classical Hodgkin Lymphoma	BC Cancer
L-591	75bp	HiSeq	Classical Hodgkin Lymphoma	BC Cancer
SUP-HD1	75bp	HiSeq	Classical Hodgkin Lymphoma	BC Cancer
U-H01	75bp	HiSeq	Classical Hodgkin Lymphoma	BC Cancer
Farage	151bp	MiSeq	Primary Mediastinal B-cell Lymphoma	BC Cancer
GRANTA519	75bp	HiSeq	Mantle Cell Lymphoma	BC Cancer
Jeko-1	75bp	HiSeq	Mantle Cell Lymphoma	BC Cancer
JVM2	75bp	HiSeq	Mantle Cell Lymphoma	BC Cancer
Mino	75bp	HiSeq	Mantle Cell Lymphoma	BC Cancer
REC-1	75bp	HiSeq	Mantle Cell Lymphoma	BC Cancer
SP-49	75bp	HiSeq	Mantle Cell Lymphoma	BC Cancer
SP-53	75bp	HiSeq	Mantle Cell Lymphoma	BC Cancer
Z138	75bp	HiSeq	Mantle Cell Lymphoma	BC Cancer
Karpas-1106P	50bp	GA	Primary Mediastinal B-cell Lymphoma	BC Cancer
MedB-1	75bp	HiSeq	Primary Mediastinal B-cell Lymphoma	BC Cancer
U-2940	75bp	HiSeq	Primary Mediastinal B-cell Lymphoma	BC Cancer
Raji	75bp	HiSeq	Burkitt’s Lymphoma	BC Cancer
Ramos	75bp	HiSeq	Burkitt’s Lymphoma	BC Cancer
DEV	50bp	GA	Nodular lymphocyte-predominant Hodgkin lymphoma	BC Cancer
DB	36bp	GA	Diffuse Large B-cell Lymphoma (Germinal Centre B-cell-like)	BC Cancer
DOHH-2	50bp	GA	Diffuse Large B-cell Lymphoma (Germinal Centre B-cell-like)	BC Cancer
HBL-1	75bp	HiSeq	Diffuse Large B-cell Lymphoma (Activated B-cell-like)	BC Cancer
HT	75bp	HiSeq	Diffuse Large B-cell Lymphoma (Germinal Centre B-cell-like)	BC Cancer
Karpas422	50bp	GA	Diffuse Large B-cell Lymphoma (Germinal Centre B-cell-like)	BC Cancer
MD903	75bp	HiSeq	Diffuse Large B-cell Lymphoma (Activated B-cell-like)	BC Cancer
NU-DHL-1	50bp	GA	Diffuse Large B-cell Lymphoma (Germinal Centre B-cell-like)	BC Cancer
NU-DUL-1	50bp	GA	Diffuse Large B-cell Lymphoma (Activated B-cell-like)	BC Cancer
OCI-Ly1	50bp	GA	Diffuse Large B-cell Lymphoma (Germinal Centre B-cell-like)	BC Cancer
OCI-Ly10	75bp	HiSeq	Diffuse Large B-cell Lymphoma (Activated B-cell-like)	BC Cancer
OCI-Ly3	75bp	HiSeq	Diffuse Large B-cell Lymphoma (Activated B-cell-like)	BC Cancer
OCI-Ly7	50bp	GA	Diffuse Large B-cell Lymphoma (Germinal Centre B-cell-like)	BC Cancer
Pfeiffer	75bp	HiSeq	Diffuse Large B-cell Lymphoma (Germinal Centre B-cell-like)	BC Cancer
SU-DHL-10	75bp	HiSeq	Diffuse Large B-cell Lymphoma (Germinal Centre B-cell-like)	BC Cancer
SU-DHL-4	75bp	HiSeq	Diffuse Large B-cell Lymphoma (Germinal Centre B-cell-like)	BC Cancer
SU-DHL-5	75bp	HiSeq	Diffuse Large B-cell Lymphoma (Germinal Centre B-cell-like)	BC Cancer
SU-DHL-6	50bp	GA	Diffuse Large B-cell Lymphoma (Germinal Centre B-cell-like)	BC Cancer
SU-DHL-8	75bp	HiSeq	Diffuse Large B-cell Lymphoma (Activated B-cell-like)	BC Cancer
Toledo	75bp	HiSeq	Diffuse Large B-cell Lymphoma (Germinal Centre B-cell-like)	BC Cancer
WSU-DLCL2	50bp	GA	Diffuse Large B-cell Lymphoma (Germinal Centre B-cell-like)	BC Cancer

RNA sequencing reads from each cell line were aligned to the hg19 reference using the STAR aligner (v2.5.2a), which also generated per-gene counts with HTSeq.

RNA sequencing data from a previously published cohort of DLBCL patients were also analyzed (Ennishi et al., 2019). Patients whose tumours were successfully assigned to a subtype by the Lymph2Cx assay (Scott et al., 2014) were retained for analysis (N = 301). This included tumours classified as germinal centre B cell-like (GCB-DLBCL; N = 171), activated B cell-like (ABC-DLBCL; N = 96), or unclassified (N = 34). Sequencing data were re-aligned to the hg19 reference to generate counts using the same method as the cell lines.

### Comparison of Ca^2+^ Channel Gene Expression

To compare the expression of the Ca^2+^ channel genes between different pathological groups, RNA sequencing counts from all samples were analyzed in R (v3.6.1) using the DESeq2 package (v1.26.0).

For cell lines, counts from all samples were read into a merged *DESeqDataSet* object, and lowly expressed genes (1 or fewer counts across all samples) were removed. The count data were then normalized using a variance-stabilizing transformation (*vst* function). Pairwise t-tests were used to compare normalized counts of numerous genes, such as Ca^2+^ channel genes of interest, between each combination of pathologies. For specific comparisons of interest, differential expression was also performed between pathological pairs (*DESeq* function), and the results for the Ca^2+^ channel genes (*p* value, adjusted *p* value (*q* value), and log2 fold change) were assessed. The same process was performed merging count data from all DLBCL patients and comparing groups by DLBCL subtype.

### Gene Set Enrichment Analysis

In order to identify Ca^2+^ channel pathways that were potentially enriched in certain pathological comparisons, the differential expression results were used to perform gene set enrichment using the GSEA tool (v4.1.0). The GSEAPreranked module was used, ranking genes by their differential expression log2 fold change and investigating specific pathways of interest (Table 2).

**TABLE 2:** BioCarta and KEGG pathways. Components of the calcineurin, calmodulin, NFAT, and Ca^2+^ signaling pathways are listed.

Pathway	Components
Calcineurin (BioCarta)	CALM1, CALM2, CALM3, CDKN1A, CYCSP35, GNAQ, LOC124827, LOC147908, MARCKS, NFATC1, NFATC2, NFATC3, NFATC4, PLCG1, PPP3CA, PPP3CB, PPP3CC, PRKCA, PRKCB, SP1, and SP3
Calmodulin (BioCarta)	CALM1, CALM2, CALM3, CAMK1, CAMK1G, CAMK2A, CAMK2B, CAMK2D, CAMK2G, CAMK4, CAMKK1, CAMKK2, CREB1, CYCSP35, LOC124827, and LOC147908
Nuclear factor of activated T cells (NFAT) (BioCarta)	ACTA1, AGT, AKT1, CALM1, CALM2, CALM3, CALR, CAMK1, CAMK1G, CAMK4, CREBBP, CSNK1A1, CTF1, CYCSP35, EDN1, ELSPBP1, F2, FGF2, FKBP1A, GATA4, GSK3B, HAND1, HAND2, HBEGF, HRAS, IGF1, LIF, LOC124827, LOC147908, MAP2K1, MAPK1, MAPK14, MAPK3, MAPK8, MEF2C, MYH2, NFATC1, NFATC2, NFATC3, NFATC4, NKX2-5, NPPA, PIK3CA, PIK3CG, PIK3R1, PPP3CA, PPP3CB, PPP3CC, PRKACB, PRKACG, PRKAR1A, PRKAR1B, PRKAR2A, PRKAR2B, RAF1, and RPS6KB1
Ca^2+^ signaling (KEGG)	ADCY1, ADCY2, ADCY3, ADCY4, ADCY7, ADCY8, ADCY9, ADORA2A, ADORA2B, ADRA1A, ADRA1B, ADRA1D, ADRB1, ADRB2, ADRB3, AGTR1, ATP2A1, ATP2A2, ATP2A3, ATP2B1, ATP2B2, ATP2B3, ATP2B4, AVPR1A, AVPR1B, BDKRB1, BDKRB2, BST1, CACNA1A, CACNA1B, CACNA1C, CACNA1D, CACNA1E, CACNA1F, CACNA1G, CACNA1H, CACNA1I, CACNA1S, CALM1, CALM2, CALM3, CALML3, CALML5, CALML6, CAMK2A, CAMK2B, CAMK2D, CAMK2G, CAMK4, CCKAR, CCKBR, CD38, CHP, CHP2, CHRM1, CHRM2, CHRM3, CHRM5, CHRNA7, CYSLTR1, CYSLTR2, DRD1, DRD5, EDNRA, EDNRB, EGFR, ERBB2, ERBB3, ERBB4, F2R, GNA11, GNA14, GNA15, GNAL, GNAQ, GNAS, GRIN1, GRIN2A, GRIN2C, GRIN2D, GRM1, GRM5, GRPR, HRH1, HRH2, HTR2A, HTR2B, HTR2C, HTR4, HTR5A, HTR6, HTR7, ITPKA, ITPKB, ITPR1, ITPR2, ITPR3, LHCGR, LOC729317, LTB4R2, MYLK, MYLK2, MYLK3, NOS1, NOS2, NOS3, NTSR1, OXTR, P2RX1, P2RX2, P2RX3, P2RX4, P2RX5, P2RX6, P2RX7, PDE1A, PDE1B, PDE1C, PDGFRA, PDGFRB, PHKA1, PHKA2, PHKB, PHKG1, PHKG2, PLCB1, PLCB2, PLCB3, PLCB4, PLCD1, PLCD3, PLCD4, PLCE1, PLCG1, PLCG2, PLCZ1, PLN, PPID, PPP3CA, PPP3CB, PPP3CC, PPP3R1, PPP3R2, PRKACA, PRKACB, PRKACG, PRKCA, PRKCB, PRKCG, PRKX, PTAFR, PTGER1, PTGER3, PTGFR, PTK2B, RYR1, RYR2, RYR3, SLC25A31, SLC25A4, SLC25A5, SLC25A6, SLC8A1, SLC8A2, SLC8A3, SPHK1, SPHK2, TACR1, TACR2, TACR3, TBXA2R, TNNC1, TNNC2, TRHR, TRPC1, VDAC1, VDAC2, and VDAC3

## Results

### ORAI Expression Varies Among Lymphoma Cell Lines

**FIGURE 1:**
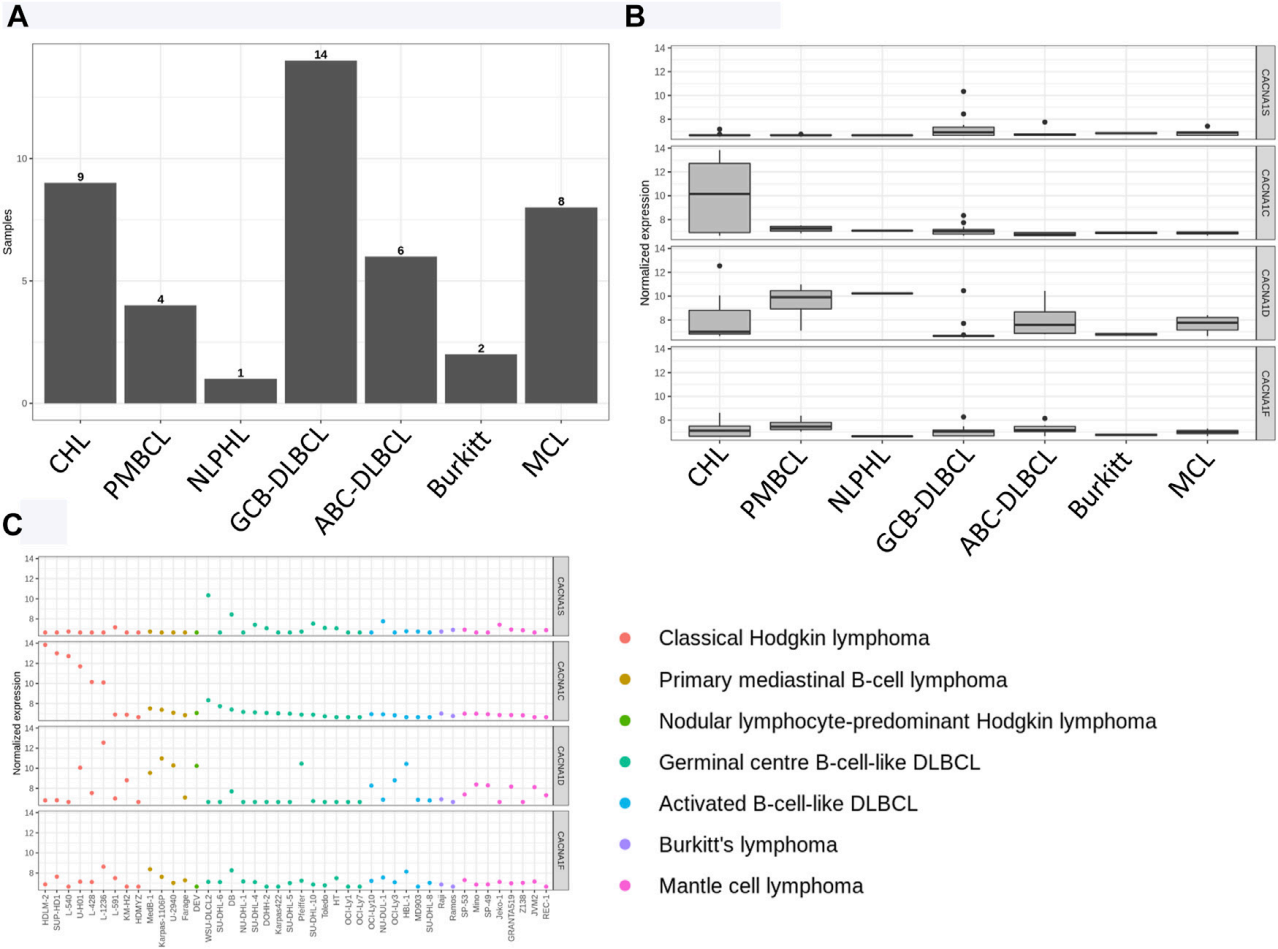
Ca_V_1 channel expression in lymphoma cell lines. **(A)** Cell lines were classified according to pathology. **(B)** Expression (normalized and log-transformed counts) of the Ca_V_1 channels (CACNA1S, Ca_V_1.1; CACNA1C, Ca_V_1.2; CACNA1D, Ca_V_1.3; CACNA1F, Ca_V_1.4). The boxes signify the interquartile range. The vertical lines correspond to the largest value that is no further than 1.5 times the interquartile range. The dots designate outliers. **(C)** Ca_V_1 channel expression is shown for each cell line.

The included cell line categories consisted of classical Hodgkin lymphoma (CHL), primary mediastinal B cell lymphoma (PMBCL), nodular lymphocyte-predominant Hodgkin lymphoma (NLPHL), GCB-DLBCL, ABC-DLBCL, Burkitt’s lymphoma, and mantle cell lymphoma (MCL) (Figure 1A). Engagement at the T cell receptor leads to Ca^2+^ release from stores, such as the endoplasmic reticulum (ER), and Ca^2+^ entry at the plasma membrane in a process known as store-operated Ca^2+^ entry (SOCE) (Omilusik et al., 2013). STIM1 and STIM2 act as ER Ca^2+^ sensors, whereas ORAI1 (as well as ORAI2 and ORAI3) is a pore-forming molecule at the cell surface (Omilusik et al., 2013). RNA sequencing data were used to perform pairwise expression comparisons between the different cell line pathologies for multiple genes of interest, including those belonging to the ORAI/STIM family. In the current study, MCL cell lines exhibited greater ORAI1 expression when compared to those representing GCB-DLBCL (adjusted *p* value = 0.028) (Supplementary Table S1). As increased expression of cyclin D1 is a feature of MCL (Vogt et al., 2017), and cyclin D1 has been linked to Ca^2+^ signaling (Kahl and Means, 2004), the high ORAI1 expression in MCL was expected. Moreover, ORAI2 showed increased expression in ABC-DLBCL cell lines relative to CHL cell lines (adjusted *p* value = 0.046) (Supplementary Table S1). Interestingly, the pairwise cell line analysis did not indicate any significant differences in expression level for STIM1/2 (Supplementary Table S1), suggesting that ER Ca^2+^ sensing might not vary to a great extent across different types of lymphoma, although this would require further investigation.

Although ORAI and STIM play important roles, other Ca^2+^ channels have been shown to be involved in lymphocyte function as well. We have previously provided evidence of voltage-dependent Ca^2+^ channel expression in the Jurkat cell line and peripheral blood T cells (Kotturi et al., 2003). Furthermore, we have previously characterized the role of Ca_V_1.4 in lymphocytes, particularly T cells, highlighting its importance in SOCE and lymphocyte immune responses (Omilusik et al., 2011). In terms of B cells, increased Ca_V_1.2 expression has been recorded for B cell childhood acute lymphoblastic leukemia and marginal zone B cell lymphoma (Wang et al., 2015). Given this, we investigated the Ca_V_1 family in the current study to assess differences in expression among various lymphoma cell lines, with the normalized expression of the four Ca_V_1 channels shown in Figures 1B,C. No statistically significant differences in Ca_V_1 channel expression were observed for the comparisons listed in Table 3. As these comparisons already had the lowest *q* values (Table 3), differences in expression for other comparisons were also deemed not significant.

**TABLE 3:** Comparisons of L-type Ca^2+^ channel expression in cell lines. Statistical analysis is presented in the form of *p* values and *q* values.

Ca_V_	Comparison with the Lowest *p* Value and q Value	Category with Higher Expression	*p* value	*q* value
1.1	GCB-DLBCL versus PMBCL	GCB-DLBCL	0.0521351928659242	0.254503787844234
1.2	ABC-DLBCL versus CHL	CHL	0.00651509770928301	0.091494633047757
1.3	Burkitt versus MCL	MCL	0.021600429461562	0.1687969044214
1.4	Burkitt versus PMBCL	PMBCL	0.0645300423866672	0.273783609609632

### CHL Exhibits Reduced Ca_V_1.1 and Higher Ca_V_1.2 Expression Levels Relative to Other Kinds of B Cell Lymphoma

**FIGURE 2:**
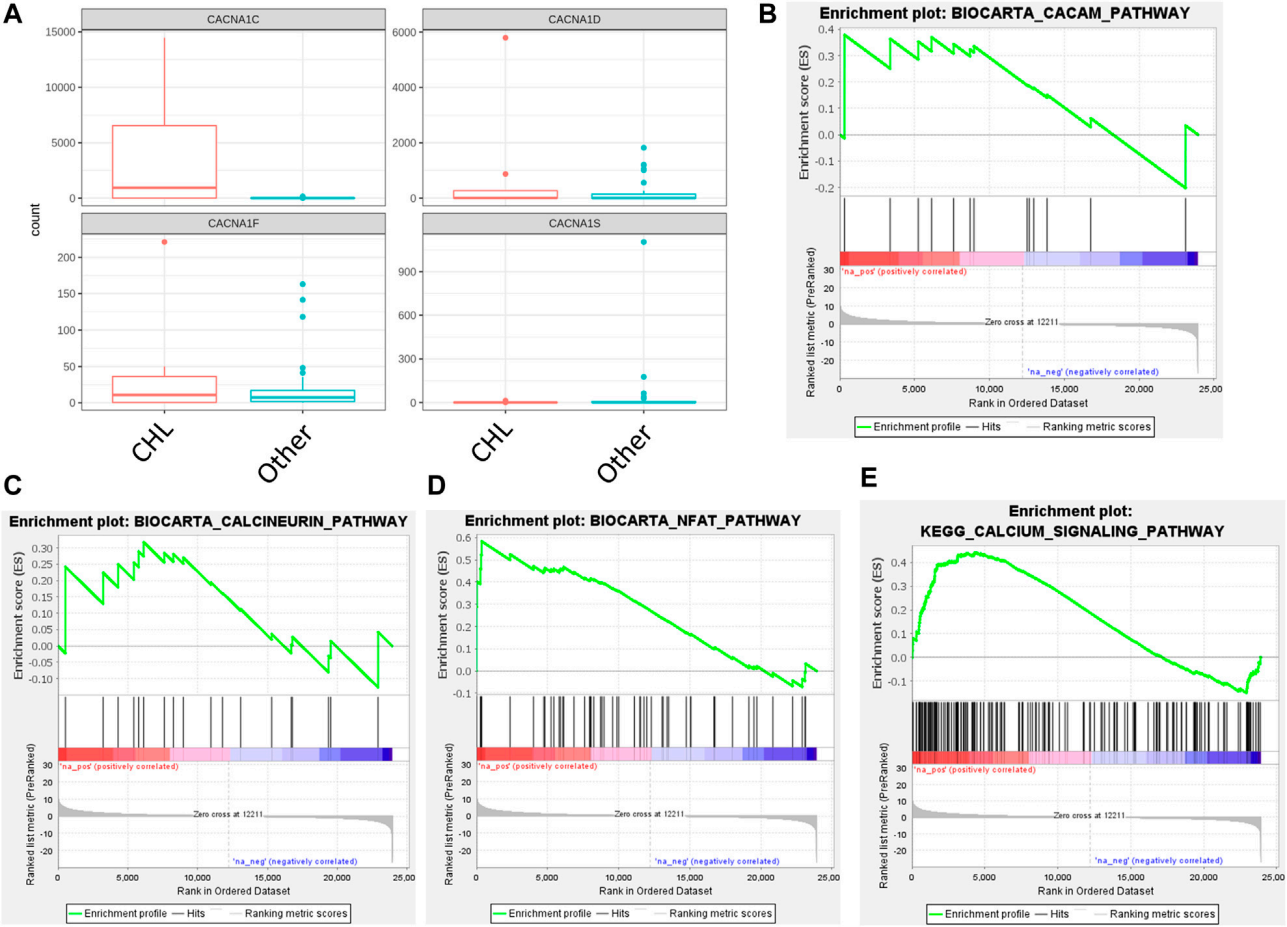
Ca_V_1 channel expression and BioCarta/KEGG pathways in CHL cell lines. **(A)** Ca_V_1 channel expression (CACNA1S, Ca_V_1.1; CACNA1C, Ca_V_1.2; CACNA1D, Ca_V_1.3; CACNA1F, Ca_V_1.4) in CHL and other B cell lymphomas. **(B–E)** Pathways for **(B)** calmodulin (BioCarta), **(C)** calcineurin (BioCarta), **(D)** NFAT (BioCarta), and **(E)** Ca^2+^ signaling (KEGG) were assessed for enrichment in the CHL cell lines relative to other varieties of B cell lymphoma.

The cell lines of the CHL category were of interest for further study due to the relatively high expression of Ca_V_1.2 (Figure 1B). Differential expression was performed between the CHL cell lines and the other B cell lymphoma cell lines to assess the expression of the L-type Ca^2+^ channels (Figure 2A). Expression of Ca_V_1.1 was significantly lower in CHL (adjusted *p* value = 0.004), and the expression of the Ca_V_1.2 channel was significantly greater in CHL (adjusted *p* value = 1.8 × 10^−16^). Differential expression also showed that the expression levels of ORAI2 (adjusted *p* value = 0.00034) and STIM2 (adjusted *p* value = 0.0016) were lower in the CHL cell lines. In order to determine whether pathways related to Ca^2+^ were affected in CHL, three pathways from BioCarta and one from the KEGG were evaluated. Although no significant enrichment was detected between CHL and other B cell lymphomas for the calcineurin and calmodulin pathways, the NFAT (nominal *p* value <0.001; FDR *q* value <0.001; FWER *p* value <0.001) and KEGG Ca^2+^ signaling (nominal *p* value <0.001; FDR q value <0.001; FWER *p* value <0.001) pathways were found to be significantly upregulated in CHL (Figures 2B–E).

Another category of cell lines that underwent more assessment was PMBCL due to the Ca_V_1.3 channel showing relatively elevated expression (Figure 1B). However, differential expression analysis of the Ca_V_1 channels found no significant differences when comparing PMBCL to other B cell lymphoma cell lines (data not shown). Similarly, differential expression did not show any differences in ORAI/STIM expression (data not shown). In PMBCL, none of the four pathways showed significant upregulation or downregulation according to GSEA (data not shown).

### Expression Profile of Ca_V_1.3 is Distinct From the Profiles of the Other Ca_V_1 Channels in DLBCL Patient Samples

**FIGURE 3:**
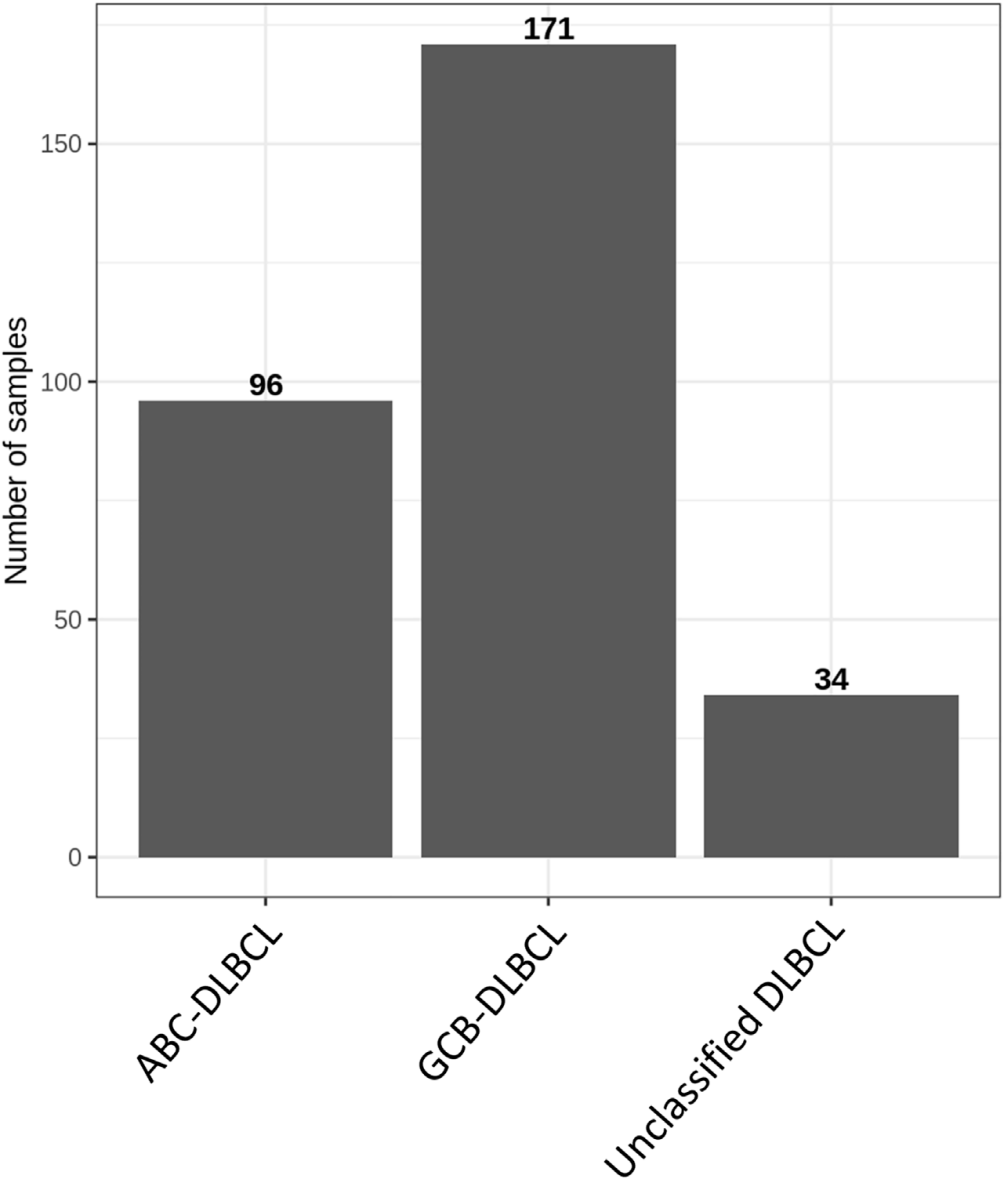
DLBCL patient cohort by cell of origin subtype. DLBCL patient samples (N = 301) were categorized as ABC-DLBCL (N = 96), GCB-DLBCL (N = 171), or unclassified DLBCL (N = 34) according to the Lymph2Cx assay.

**FIGURE 4:**
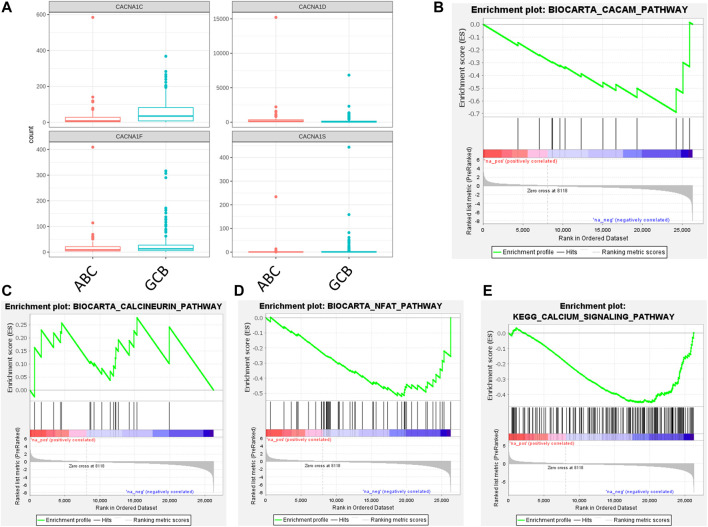
Ca_V_1 channel expression and BioCarta/KEGG pathways in ABC-DLBCL and GCB-DLBCL patient samples. **(A)** Ca_V_1 channel expression (CACNA1S, Ca_V_1.1; CACNA1C, Ca_V_1.2; CACNA1D, Ca_V_1.3; CACNA1F, Ca_V_1.4) in ABC-DLBCL and GCB-DLBCL. **(B–E)** Differential expression results were assessed for pathway enrichment of **(B)** calmodulin (BioCarta), **(C)** calcineurin (BioCarta), **(D)** NFAT (BioCarta), and **(E)** Ca^2+^ signaling (KEGG).

Aside from cell lines, the study of samples from patients with DLBCL was also conducted. Cell of origin was defined for patient samples, assigning each to ABC-DLBCL, GCB-DLBCL, or unclassified DLBCL (Figure 3). Numerous genes were evaluated for their expression levels with the use of RNA sequencing data, including the ORAI/STIM family. No differences in ORAI/STIM expression were observed from the pairwise comparisons (Table 4). To further assess the differences associated with DLBCL cell of origin, differential expression analysis was performed between ABC-DLBCL and GCB-DLBCL patient samples and used to assess the four Ca_V_1 channels (Figure 4A). GCB-DLBCL demonstrated higher expression of Ca_V_1.1 (adjusted *p* value = 2.2 × 10^−10^), Ca_V_1.2 (adjusted *p* value = 2.5 × 10^−6^), and Ca_V_1.4 (adjusted *p* value = 0.0013). In contrast, ABC-DLBCL displayed greater expression of Ca_V_1.3 (adjusted *p* value = 0.0016). Differential expression analysis of the patient samples did not indicate any significant differences in ORAI/STIM expression between ABC-DLBCL and GCB-DLBCL (data not shown). For pathways associated with Ca^2+^, the calcineurin and NFAT pathways were unchanged between GCB-DLBCL and ABC-DLBCL (Figures 4C,D). However, the calmodulin (nominal *p* value = 0.037; FDR q value = 0.037; FWER *p* value = 0.032) and KEGG (nominal *p* value = 0.04; FDR q value = 0.04; FWER *p* value = 0.04) pathways were both downregulated in ABC-DLBCL (Figures 4B,E).

**TABLE 4:** Comparisons of ORAI/STIM expression in DLBCL patient samples. The *p* value and *q* value for each comparison are shown.

Gene	COO A	COO B	Mean A	Mean B	*p* value	q value
ORAI1	GCB	ABC	7.71096066894052	7.83107649954498	0.208403954574778	0.41883124851436
	GCB	Unclassified	7.71096066894052	7.82500105710116	0.32273153927664	0.538753456695682
	ABC	Unclassified	7.83107649954498	7.82500105710116	0.962399139380119	0.986380548158669
ORAI2	ABC	Unclassified	11.4239520360561	11.0771101686768	0.0756808552933548	0.246707670011409
	GCB	ABC	11.2701801394973	11.4239520360561	0.219636549829334	0.428912885044076
	GCB	Unclassified	11.2701801394973	11.0771101686768	0.299007694731689	0.520122628650921
ORAI3	GCB	ABC	9.38887758513438	9.29082762801457	0.143491212884708	0.343481033490216
	ABC	Unclassified	9.29082762801457	9.43745538376645	0.180810083575845	0.396852604528146
	GCB	Unclassified	9.38887758513438	9.43745538376645	0.638326098387733	0.808155977775295
STIM1	GCB	ABC	10.2794800129143	10.1798834785399	0.314227244848016	0.53315606297983
	ABC	Unclassified	10.1798834785399	10.3018108011769	0.422786360206746	0.651958096371479
	GCB	Unclassified	10.2794800129143	10.3018108011769	0.87562962451273	0.959022922085371
STIM2	ABC	Unclassified	10.6677890074025	10.5761563926166	0.52196480309146	0.738890716785782
	GCB	Unclassified	10.6595800398733	10.5761563926166	0.56420482060024	0.766037193868797
	GCB	ABC	10.6595800398733	10.6677890074025	0.921572084819698	0.973292967131008

## Discussion

As a range of lymphocyte functions are affiliated with Ca^2+^, such as proliferation and activation (Omilusik et al., 2013), a study of Ca^2+^ channel expression and signaling pathway expression was conducted in an attempt to disentangle some of the complexities of lymphoma. The results presented here refer to cell lines and DLBCL patient samples, with a discussion regarding each of these groups being provided below. Methods have been used previously (Aoki et al., 2020) to reaffirm the expression pattern of certain genes; however, a notable limitation of the current study is that no such validation has been completed.

The CHL cell lines included in this study consisted of the HDLM-2, HDMYZ, KM-H2, L-1236, L-428, L-540, L-591, SUP-HD1, and U-H01 cell lines (Table 1). Differential expression analysis revealed that Ca_V_1.2 was more highly expressed in CHL cell lines relative to cell lines belonging to other types of B cell lymphoma. Ca_V_1.2 has been linked to interleukin-13 (IL-13) in the context of T helper 2 (T_H_2) cells in that IL-13 levels drop in response to Ca_V_1.2 knockdown (Robert et al., 2014). IL-13 has been shown to be expressed by cell lines of Hodgkin lymphoma (Kapp et al., 1999). Furthermore, in a study published soon afterward (Skinnider et al., 2001), it was shown that most CHL patients exhibited expression of IL-13, whereas this trait was either observed in none or fewer than 50% of patients with other forms of lymphoma, such as DLBCL. Signal transducer and activator of transcription 6 (STAT6), which is activated by IL-13, was found to be phosphorylated in cell lines of Hodgkin lymphoma (Skinnider et al., 2002). Therefore, Ca_V_1.2 could possibly be playing a role in the regulation of IL-13 and STAT6 in CHL. Since the NFAT and KEGG Ca^2+^ signaling pathways (Table 2) were shown to be upregulated in the CHL cell lines, it is also possible that Ca_V_1.2 influences the components of these pathways. In contrast to Ca_V_1.2, reduced expression of ORAI2 and STIM2 was noted in the CHL cell lines via differential expression analysis. Given that STIM1 is known to activate ORAI channels and hinder Ca_V_1.2 (Wang et al., 2010), it was surprising that the differential expression analysis in the current study did not show a significant reduction in STIM1 expression in the CHL cell lines. As STIM1 and STIM2 differ in their expression profiles (at least in T cells) and the ER Ca^2+^ concentration to which they respond (Shaw and Feske, 2012), this could potentially explain why only one of them, STIM2, showed a change in expression between CHL and other B cell lymphomas.

Among the GCB-DLBCL and ABC-DLBCL patients, the expression of the Ca_V_1.3 channel was found to be higher in ABC-DLBCL by differential expression. It is known that L-type Ca_V_ channel constraint leads to reduced function of the transcription factor nuclear factor kappa B (NF-κB) (Lilienbaum and Israël, 2003). Within the promoter for the *bcl-2* gene, which encodes the B cell leukemia/lymphoma-2 (Bcl-2) “anti-apoptosis” protein, there exists at least one binding site for NF-κB (Catz and Johnson, 2001). BCL2, as part of a network of genes, was deemed to be upregulated in ABC-DLBCL (Blenk et al., 2007). Another component of this network that was ascertained as being upregulated in the ABC kind of DLBCL was IRF4 (Blenk et al., 2007), and the promoter for this gene is known to have NF-κB binding sites as well (Sharma et al., 2002). Future studies could potentially further characterize these components in terms of how they might functionally relate to the Ca_V_1.3 Ca^2+^ channel in the specific context of DLBCL, particularly since no differences in ORAI/STIM expression were detected in the patient samples in our study. This latter finding suggests that ORAI channels might not be contributing to differences in DLBCL phenotype in patients and that other Ca^2+^ channels, such as Ca_V_1.3, could be playing this role instead. Furthermore, the calmodulin and KEGG Ca^2+^ signaling pathways (Table 2) were downregulated in ABC-DLBCL relative to GCB-DLBCL, suggesting that these pathways could also be linked to differences in DLBCL phenotype. The DLBCL patient sample RNA sequencing did not cover control B cells due to a lack of normal matches at the time. Investigation of Ca_V_ channels from this perspective would potentially be valuable as well. In order to validate these suggested mechanisms linked to Ca^2+^, follow-up experiments would need to be conducted.

To conclude, the L-type Ca_V_ channels represent a potential target class in the field of anti-lymphoma therapeutics. By targeting specific Ca_V_1 channels and, by extension, the mechanisms associated with each channel, this could lead to updated strategies to treat lymphoma.

## Data Availability

RNA sequencing data for the lymphoma cell lines generated at BC Cancer are available via controlled access through GEO (accession number GSE189927). Data for the DLBCL patients were previously reported, and information on the accession is available in the original article (Ennishi et al., 2019).
