# Supplementary material for: Distinct Gene Expression Patterns of Calcium Channels and Related Signaling Pathways Discovered in Lymphomas
Source: Front Pharmacol. 2022 May 24;13:795176. doi: 10.3389/fphar.2022.795176 (PMC9172636; doi:10.3389/fphar.2022.795176)
Supplement: Supplementary file 1 [file Table1.docx]

**Table S1. Comparisons of ORAI/STIM expression between cell line pathologies**

| **Gene** | **Pathology A** | **Pathology B** | **Mean A** | **Mean B** | **p value** | **q value** |
| --- | --- | --- | --- | --- | --- | --- |
| ORAI1 | GCB-DLBCL | MCL | 7.92137076925926 | 8.70984299969743 | 0.000199073551088646 | 0.0278999158024612 |
|  | Burkitt | GCB-DLBCL | 8.64345922225998 | 7.92137076925926 | 0.0270748562966009 | 0.187396683938616 |
|  | CHL | GCB-DLBCL | 8.5976413319758 | 7.92137076925926 | 0.0376279755750542 | 0.221129654397152 |
|  | ABC-DLBCL | GCB-DLBCL | 8.44089888323384 | 7.92137076925926 | 0.0933854758496186 | 0.316440523662842 |
|  | MCL | PMBCL | 8.70984299969743 | 8.29760732970939 | 0.28556469093273 | 0.511482782835149 |
|  | GCB-DLBCL | PMBCL | 7.92137076925926 | 8.29760732970939 | 0.326774404624398 | 0.547149069444951 |
|  | ABC-DLBCL | MCL | 8.44089888323384 | 8.70984299969743 | 0.344733027836001 | 0.548134626670455 |
|  | Burkitt | PMBCL | 8.64345922225998 | 8.29760732970939 | 0.370867862051682 | 0.558697461341725 |
|  | CHL | PMBCL | 8.5976413319758 | 8.29760732970939 | 0.482780105615466 | 0.640421507070518 |
|  | ABC-DLBCL | Burkitt | 8.44089888323384 | 8.64345922225998 | 0.494656237693713 | 0.649487661687274 |
|  | ABC-DLBCL | CHL | 8.44089888323384 | 8.5976413319758 | 0.664875759583279 | 0.779038223743891 |
|  | CHL | MCL | 8.5976413319758 | 8.70984299969743 | 0.699306584467215 | 0.804783943407044 |
|  | ABC-DLBCL | PMBCL | 8.44089888323384 | 8.29760732970939 | 0.728092082538465 | 0.819832037271285 |
|  | Burkitt | MCL | 8.64345922225998 | 8.70984299969743 | 0.739588910504185 | 0.827553873300872 |
|  | Burkitt | CHL | 8.64345922225998 | 8.5976413319758 | 0.880002382468802 | 0.920866423987331 |
| ORAI2 | ABC-DLBCL | CHL | 12.3028874373236 | 10.0796884827888 | 0.0013647133641262 | 0.0458519497914637 |
|  | CHL | MCL | 10.0796884827888 | 12.3650346642485 | 0.0018383037981028 | 0.0536829185870762 |
|  | CHL | GCB-DLBCL | 10.0796884827888 | 11.9467207604036 | 0.00723383882077477 | 0.0973118001146357 |
|  | Burkitt | CHL | 12.8485696890308 | 10.0796884827888 | 0.0114048631904642 | 0.127026579673101 |
|  | CHL | PMBCL | 10.0796884827888 | 11.671008463125 | 0.0333177049928117 | 0.206954206013042 |
|  | Burkitt | PMBCL | 12.8485696890308 | 11.671008463125 | 0.147817199832816 | 0.396962428908373 |
|  | Burkitt | GCB-DLBCL | 12.8485696890308 | 11.9467207604036 | 0.213229011245777 | 0.443353579960381 |
|  | ABC-DLBCL | PMBCL | 12.3028874373236 | 11.671008463125 | 0.240511028615058 | 0.470343464637723 |
|  | MCL | PMBCL | 12.3650346642485 | 11.671008463125 | 0.260551529256689 | 0.487507447237997 |
|  | ABC-DLBCL | GCB-DLBCL | 12.3028874373236 | 11.9467207604036 | 0.400888402240491 | 0.570846233315262 |
|  | ABC-DLBCL | Burkitt | 12.3028874373236 | 12.8485696890308 | 0.413603018848561 | 0.582954654929826 |
|  | GCB-DLBCL | MCL | 11.9467207604036 | 12.3650346642485 | 0.450568068329002 | 0.612922448646412 |
|  | Burkitt | MCL | 12.8485696890308 | 12.3650346642485 | 0.460602364815312 | 0.621188157976391 |
|  | GCB-DLBCL | PMBCL | 11.9467207604036 | 11.671008463125 | 0.648318409143642 | 0.768436108411852 |
|  | ABC-DLBCL | MCL | 12.3028874373236 | 12.3650346642485 | 0.877768289927821 | 0.919521592367631 |
| ORAI3 | ABC-DLBCL | Burkitt | 9.81254356264722 | 9.22004522971868 | 0.501941013305423 | 0.652860190460342 |
|  | Burkitt | MCL | 9.22004522971868 | 9.80945511974473 | 0.50333245041549 | 0.653354513667261 |
|  | Burkitt | CHL | 9.22004522971868 | 9.71924559061332 | 0.561012812413618 | 0.702807259507169 |
|  | ABC-DLBCL | GCB-DLBCL | 9.81254356264722 | 9.63090249325952 | 0.608553414032281 | 0.738957717039199 |
|  | Burkitt | GCB-DLBCL | 9.22004522971868 | 9.63090249325952 | 0.623309049661656 | 0.748898287814191 |
|  | Burkitt | PMBCL | 9.22004522971868 | 9.66496467912171 | 0.632472974965433 | 0.755692124218871 |
|  | GCB-DLBCL | MCL | 9.63090249325952 | 9.80945511974473 | 0.633693862746944 | 0.756235195676911 |
|  | CHL | GCB-DLBCL | 9.71924559061332 | 9.63090249325952 | 0.804071014379231 | 0.878807479403059 |
|  | ABC-DLBCL | CHL | 9.81254356264722 | 9.71924559061332 | 0.817058887387176 | 0.884291971442248 |
|  | ABC-DLBCL | PMBCL | 9.81254356264722 | 9.66496467912171 | 0.822419847890696 | 0.887938532151626 |
|  | MCL | PMBCL | 9.80945511974473 | 9.66496467912171 | 0.828656662033212 | 0.891196787469681 |
|  | CHL | MCL | 9.71924559061332 | 9.80945511974473 | 0.830489485199326 | 0.892672558134384 |
|  | CHL | PMBCL | 9.71924559061332 | 9.66496467912171 | 0.934265148230633 | 0.957673334299843 |
|  | GCB-DLBCL | PMBCL | 9.63090249325952 | 9.66496467912171 | 0.957033058570763 | 0.972321567297364 |
|  | ABC-DLBCL | MCL | 9.81254356264722 | 9.80945511974473 | 0.994101214462413 | 0.99512817852694 |
| STIM1 | ABC-DLBCL | CHL | 10.497229577526 | 11.5911786553108 | 0.0819301704616285 | 0.301291594600827 |
|  | ABC-DLBCL | GCB-DLBCL | 10.497229577526 | 11.4660980508947 | 0.111721764744479 | 0.348352871113368 |
|  | CHL | MCL | 11.5911786553108 | 11.1857260563232 | 0.156644651687224 | 0.406726882976073 |
|  | ABC-DLBCL | Burkitt | 10.497229577526 | 11.3174238973457 | 0.197235078809842 | 0.427563291648182 |
|  | ABC-DLBCL | MCL | 10.497229577526 | 11.1857260563232 | 0.223045679914989 | 0.45549265297708 |
|  | ABC-DLBCL | PMBCL | 10.497229577526 | 11.3859178885813 | 0.245828629457749 | 0.476892776665784 |
|  | GCB-DLBCL | MCL | 11.4660980508947 | 11.1857260563232 | 0.26151602285562 | 0.487658598244322 |
|  | Burkitt | CHL | 11.3174238973457 | 11.5911786553108 | 0.513990757119204 | 0.664076058198011 |
|  | CHL | GCB-DLBCL | 11.5911786553108 | 11.4660980508947 | 0.700419743779535 | 0.805586625189755 |
|  | Burkitt | GCB-DLBCL | 11.3174238973457 | 11.4660980508947 | 0.706041569477567 | 0.807597659868597 |
|  | Burkitt | MCL | 11.3174238973457 | 11.1857260563232 | 0.721955092291732 | 0.816782818950016 |
|  | MCL | PMBCL | 11.1857260563232 | 11.3859178885813 | 0.724320397035895 | 0.818980705633351 |
|  | CHL | PMBCL | 11.5911786553108 | 11.3859178885813 | 0.73175989635705 | 0.821640022676688 |
|  | GCB-DLBCL | PMBCL | 11.4660980508947 | 11.3859178885813 | 0.891033593179736 | 0.927092648189946 |
|  | Burkitt | PMBCL | 11.3174238973457 | 11.3859178885813 | 0.911639474183073 | 0.942270560515625 |
| STIM2 | CHL | GCB-DLBCL | 10.1202047246013 | 11.4209671969562 | 0.00316695391033485 | 0.0635443927169868 |
|  | GCB-DLBCL | MCL | 11.4209671969562 | 10.2440013104848 | 0.0054150130642253 | 0.0819866821755362 |
|  | ABC-DLBCL | CHL | 11.7665517615493 | 10.1202047246013 | 0.017771555533465 | 0.155842871601155 |
|  | ABC-DLBCL | MCL | 11.7665517615493 | 10.2440013104848 | 0.0246719404371036 | 0.179752708898897 |
|  | CHL | PMBCL | 10.1202047246013 | 11.0241453022958 | 0.160644599970422 | 0.410597626963188 |
|  | MCL | PMBCL | 10.2440013104848 | 11.0241453022958 | 0.209949182858851 | 0.443226052702018 |
|  | Burkitt | CHL | 11.7622535850115 | 10.1202047246013 | 0.292978193012431 | 0.518254230083982 |
|  | ABC-DLBCL | PMBCL | 11.7665517615493 | 11.0241453022958 | 0.315467993812141 | 0.540563193640963 |
|  | Burkitt | MCL | 11.7622535850115 | 10.2440013104848 | 0.317726194401846 | 0.541705778448096 |
|  | GCB-DLBCL | PMBCL | 11.4209671969562 | 11.0241453022958 | 0.527532513087074 | 0.676339771422229 |
|  | Burkitt | PMBCL | 11.7622535850115 | 11.0241453022958 | 0.542159335810625 | 0.68763402670222 |
|  | ABC-DLBCL | GCB-DLBCL | 11.7665517615493 | 11.4209671969562 | 0.57259487027669 | 0.710883317486371 |
|  | CHL | MCL | 10.1202047246013 | 10.2440013104848 | 0.623738616861492 | 0.74895008641733 |
|  | Burkitt | GCB-DLBCL | 11.7622535850115 | 11.4209671969562 | 0.759973929265547 | 0.842579791142237 |
|  | ABC-DLBCL | Burkitt | 11.7665517615493 | 11.7622535850115 | 0.996958343131544 | 0.99747303510012 |
